# Transforming Growth Factor-Beta Promotes Rhinovirus Replication in Bronchial Epithelial Cells by Suppressing the Innate Immune Response

**DOI:** 10.1371/journal.pone.0044580

**Published:** 2012-09-06

**Authors:** Nicole Bedke, David Sammut, Ben Green, Valia Kehagia, Patrick Dennison, Gisli Jenkins, Amanda Tatler, Peter H. Howarth, Stephen T. Holgate, Donna E. Davies

**Affiliations:** 1 Academic Unit of Clinical and Experimental Sciences, University of Southampton Faculty of Medicine, University Hospital Southampton, Southampton, United Kingdom; 2 National Institute for Health Research, Respiratory Biomedical Research Unit, University Hospital Southampton, Southampton, United Kingdom; 3 University of Nottingham, Clinical Sciences Building, Nottingham City Hospital, Nottingham, United Kingdom; National Jewish Health, United States of America

## Abstract

Rhinovirus (RV) infection is a major cause of asthma exacerbations which may be due to a deficient innate immune response in the bronchial epithelium. We hypothesized that the pleiotropic cytokine, TGF-β, influences interferon (IFN) production by primary bronchial epithelial cells (PBECs) following RV infection. Exogenous TGF-β_2_ increased RV replication and decreased IFN protein secretion in response to RV or double-stranded RNA (dsRNA). Conversely, neutralizing TGF-β antibodies decreased RV replication and increased IFN expression in response to RV or dsRNA. Endogenous TGF-β_2_ levels were higher in conditioned media of PBECs from asthmatic donors and the suppressive effect of anti-TGF-β on RV replication was significantly greater in these cells. Basal SMAD-2 activation was reduced when asthmatic PBECs were treated with anti-TGF-β and this was accompanied by suppression of SOCS-1 and SOCS-3 expression. Our results suggest that endogenous TGF-β contributes to a suppressed IFN response to RV infection possibly via SOCS-1 and SOCS-3.

## Introduction

Asthma is a chronic inflammatory disease, characterized by wheezing and bronchial hyperresponsiveness [1]; [2]. Human rhinovirus (RV) infection is a major cause of asthma exacerbations both in children and in adults worldwide [3]. Infection of epithelial cells with RV leads to the initiation of the innate immune response involving type I and type III interferons (IFNs), and expression of proinflammatory cytokines. Binding of IFNs to their receptors can occur in an autocrine or paracrine fashion, activating the JAK-STAT pathway to induce expression of more IFNs, stimulate the cellular antiviral machinery, and cause apoptosis of infected cells to limit spread of the viral infection. Previous studies have shown that primary bronchial epithelial cells (PBECs) from asthmatic patients produce significantly lower levels of IFN-β and IFN-λ in response to RV infection when compared to PBECs obtained from non-asthmatic volunteers [4]; [5]. This effect was associated with increased viral replication in and enhanced cytopathic cell death of the asthmatic cells [4].

The transforming growth factor beta (TGF-β) cytokine family has pleiotropic effects [6] including potent anti-inflammatory and profibrogenic activities which have been linked to airway remodelling in asthma [7]; [8]. TGF-β_1_ and TGF-β_2_ are produced by a variety of cells in asthmatic airways, including eosinophils [9] and bronchial epithelial cells [10], respectively. It has been suggested that, in asthma, persistent epithelial damage leads to a chronic wound scenario associated with sustained release of TGF-β_2_ and activation of subepithelial fibroblasts leading to drive airway remodelling [10]; [11]. In studies of viral infection, exogenous TGF-β has been reported to markedly increase replication of respiratory syncytial virus (RSV) in PBECs from healthy donors via a mechanism involving decreased cellular metabolism which reduced the competition for substrates during viral replication [12]. RSV is an enveloped virus which causes lower respiratory tract infections in infants and, like RV, has been implicated in asthma exacerbations [13]. More recently, treatment of bronchial fibroblasts with exogenous TGF-β_1_ to induce myofibroblast differentiation was also found to promote RV replication and this was linked to decreased IFN gene expression [14]. Since epithelial expression of TGF-β isoforms is increased in asthma [8]; [15], we hypothesized that endogenous production of TGF-β by asthmatic PBECs contributes to their lower innate immune response to RV infection. Therefore, we have investigated whether neutralization of endogenous TGF-β in cultures from asthmatic donors reduced viral replication. Conversely, we also investigated whether treatment of PBECs from non-asthmatic volunteers with exogenous TGF-β_2_ resulted in increased viral replication in association with a reduced IFN response.

## Methods

### Ethics Statement

Ethical approval for this study was obtained from the Southampton and South West Hampshire Research Ethics Committees (A), reference number 05/Q1702/165. Written informed consent was received from all normal and asthmatic donors who participated in the study.

**Figure 1:**
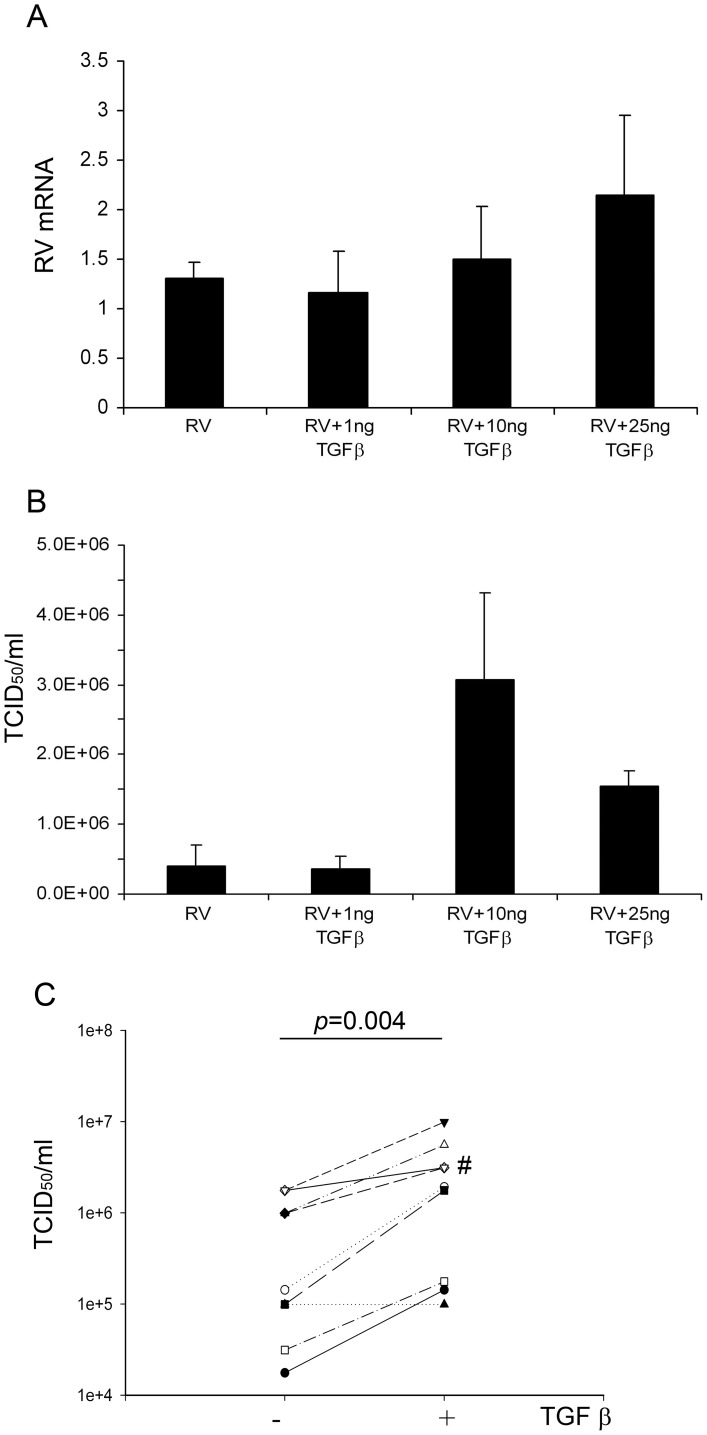
The effect of exogenous TGF-β_2_ on RV replication. PBECs from 3 non-asthmatic volunteers were pre-incubated with 0, 1, 10, and 25 ng/ml of TGF- β_2_ for 24 h, followed by infection with RV1B at 5000 TCID_50_ units/10^5^ cells. Cells were then further incubated for 48 h in the presence or absence of TGF-β_2_, as indicated. Viral replication at 24 h was measured as vRNA by RT-qPCR (A) and at 48 h by release of infectious virions into culture supernatants by TCID_50_ assays (B). The graph (C) shows data for infectious virus release from PBECs from 10 non-asthmatic donors treated without or with 10 ng/ml TGF-β_2_, followed by infection with RV1B at 5000 TCID_50_ units/10^5^ cells for 48 hours. Statistical comparison was made using a Wilcoxon rank sum test. The # mark in C indicates where 2 data points overlap (1.8e^6^→3.1e^6^ TCID_50_ units/ml).

### Generation and Titration of Human RV1B

RV1B (a gift from Professor Sebastian L. Johnston, Imperial College, London) was propagated by infecting monolayer cultures of Ohio HeLa cells (obtained from the American Type Culture Collection). Viral titers in RV1B stocks and BEC supernatants were determined by 50% tissue culture infective dose (TCID_50_) using Ohio HeLa cells, as described previously [4]. Inactivated RV1B control was prepared by exposure to UV light at 1200 mJ/cm^2^ on ice for 50 min and stored in aliquots at −80°C.

**Figure 2:**
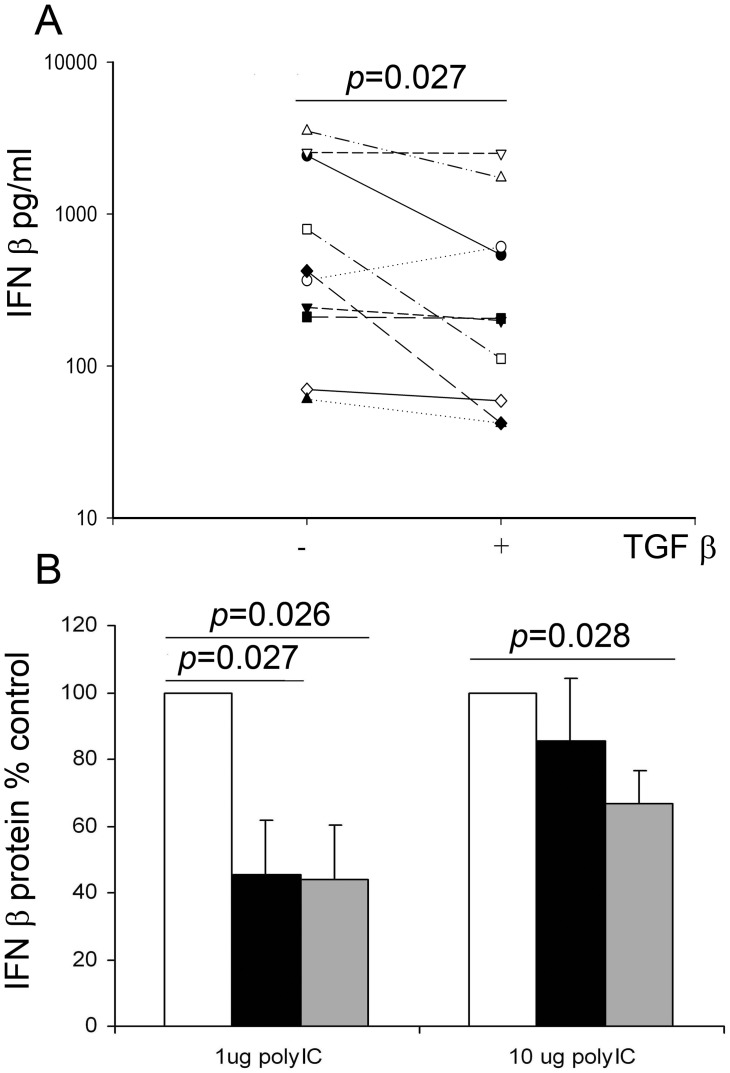
Exogenous TGF-β_2_ suppresses IFN-β release from virally infected (A) or poly IC exposed (B) PBEC cultures from non-asthmatic donors. PBEC cultures were infected with RV1B (5000 TCID_50_ units/10^5^ cells) (n = 10) or treated with poly IC (n = 5) in the presence or absence of TGF-β_2_ which was used at 1 (black bars in B) or 10 ng/ml (panel A and grey bars in B). Culture supernatants were harvested 48 hours p.i (A) or 8 h post stimulation (B) and IFN-β protein levels were measured by ELISA. In B, the data are expressed as a % of control cultures treated with poly IC in the absence of TGF-β_2_ (median (IQR) IFN-β release  = 346 (1135) and 369 (1390) pg/ml for cells treated with 1 or 10 µg/ml Poly IC, respectively. The data were analyzed using Wilcoxon’s rank sum test (A) or using a paired t-test for normally distributed data (B).

### Establishment of Primary Bronchial Epithelial Cells from Bronchial Brushings and Infection with RV1B

Bronchial brushings were obtained from normal (n = 24) and asthmatic (n = 35) donors at fibreoptic bronchoscopy following ethical approval and informed consent. The non-asthmatic subjects (M:F 9:15, mean age 27.2 (range 20–55)) had a mean (SD) FEV_1_ of 103.44 (11.87) % predicted and PC_20_ methacholine >16 mg/ml, whilst the asthmatic subjects (M:F 14:21, mean age 40.2 (range 19–70)) had an FEV_1_ of 85.7 (23.0) % predicted. The asthmatic donors were either on β_2_-agonists as sole therapy (n = 10, geometric mean PC20 2.55 mg/ml, range 0.07–17, or treated with inhaled steroids, long-acting beta-agonists plus other therapy [n = 25, no PC_20_ available]).

**Figure 3:**
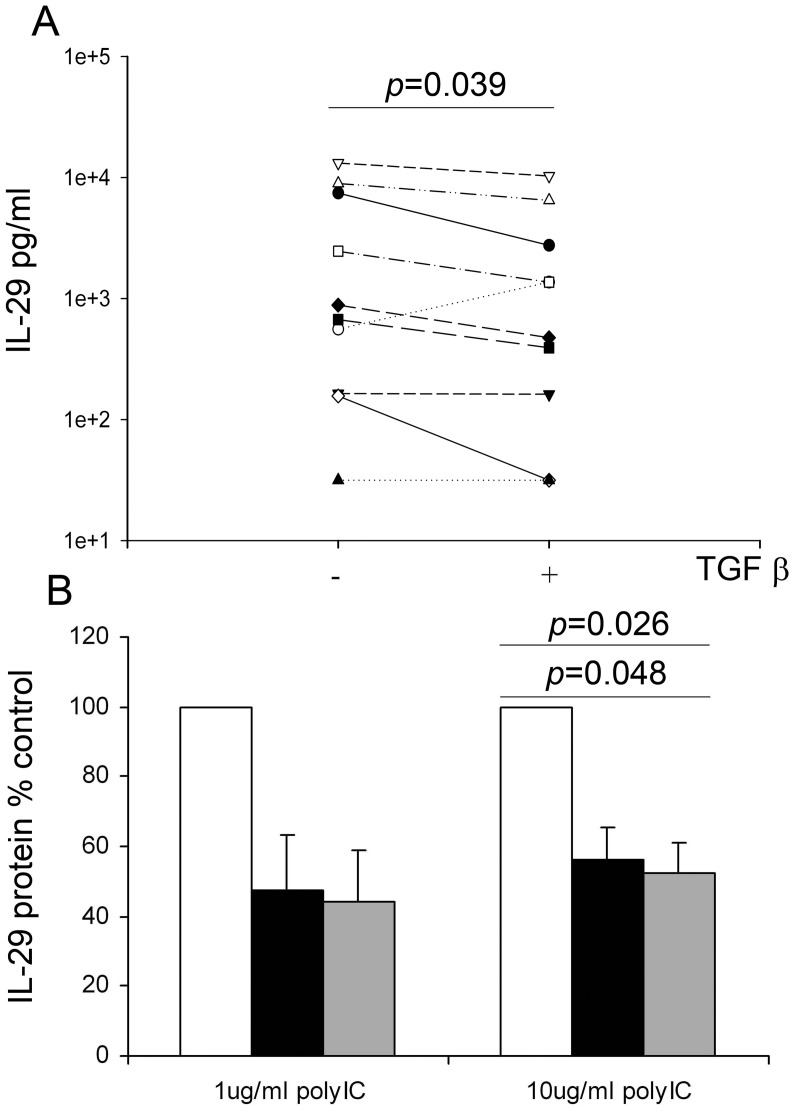
Exogenous TGF-β_2_ suppresses IFN-λ1/IL-29 release from virally infected (A) (n = 10) or poly IC (n = 4) exposed (B) PBEC cultures from non-asthmatic donors. IFNλ1/IL-29 protein levels were measured by ELISA from RV-infected or poly IC exposed PBECs treated with TGF-β_2_ as described in Figure 2. Median (IQR) IFN-λ_1_ release  = 3896 (2766) and 4932 (4941) pg/ml for cells treated with 1 or 10 µg/ml Poly IC, respectively.

Bronchial brushings were cultured until confluent in Bronchial Epithelial Growth Media (BEGM, Cambrex, Switzerland), as previously described [4], and were then seeded into 12-well plates at a cell density 0.4×10^5^ cells per well. Once cells reached 90% confluence, they were placed in BEBM basal medium (Cambrex, Switzerland), supplemented with 0.1% (w/v) bovine serum albumin (BSA) and 1% (v/v) insulin/transferrin/sodium selenite (ITS from Sigma, Poole, UK) for 24 h. Cells were then infected with RV1B at 1000 or 5000 TCID_50_ units/10^5^ cells for 1 h at room temperature with shaking. The lower innoculum of virus was applied to asthmatic PBECs to take into account their increased susceptibility to RV infection [4]. After infection, cells were washed and incubated with fresh media for the appropriate times at 37°C. Where appropriate, TGF-β_2_ (Peprotech), an anti-TGF-β mouse monoclonal antibody (MAB1835, R&D Systems, Abingdon, UK) that recognizes human TGF-β_1_ and TGF-β_2_, or an IgG isotype control antibody (both at 10 µg/ml) was added 24 h prior to infection and replaced after infection. Synthetic double stranded RNA (polyinosinic polycytidilic acid, poly IC) (Autogen Bioclear, Calne, Wiltshire, U.K) was diluted in cell culture medium before use.

**Figure 4:**
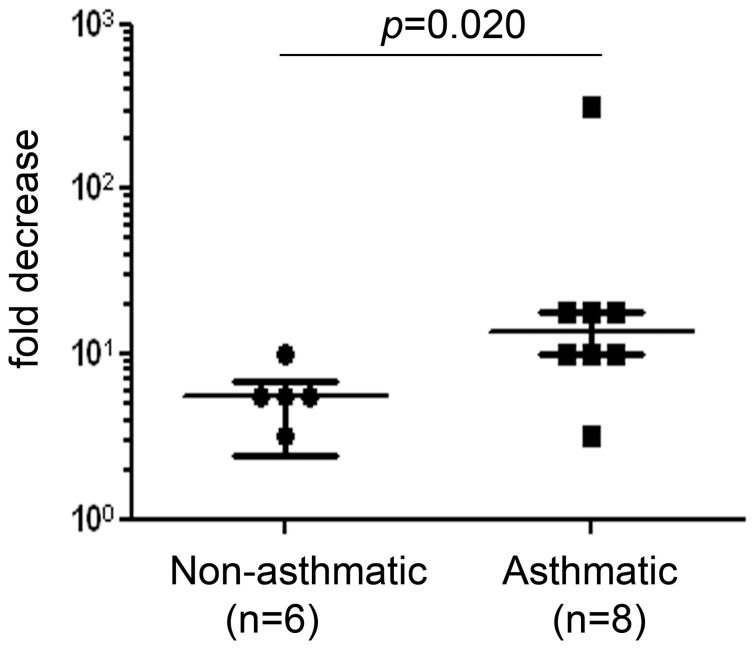
The effect of neutralizing endogenous TGF-β on RV replication. PBECs from 8 asthmatic donors or 6 non-asthmatic control subjects were pretreated for 24 h in the presence of a neutralizing anti pan TGF-β antibody or isotype control antibody before infection with RV1B (1000 units/10^5^ cells) for 48 h. The fold-decrease in viral replication by the neutralizing antibody was plotted as a ratio of the TCID_50_/ml of antibody-treated versus isotype controls. The figure shows median and interquartile range, with individual data points superimposed. Data were analysed using a Mann Whitney U test.

**Figure 5:**
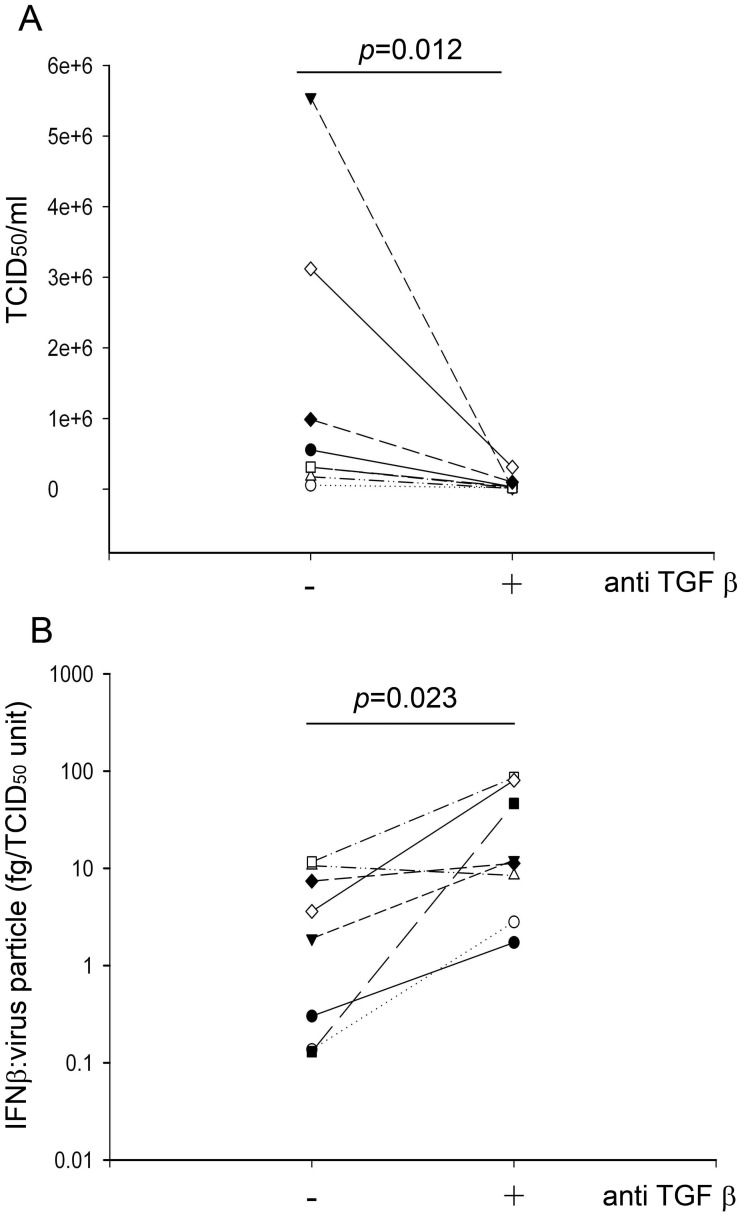
Suppression of viral replication in PBECs from asthmatic donors by neutralization of endogenous TGF-β. PBECs from asthmatic donors were pretreated for 24 h in the presence of a neutralizing anti pan TGF-β antibody or isotype control antibody before infection with RV1B (1000 units/10^5^ cells) for 48 h. In A, viral titre was determined as TCID_50_/ml using culture supernatants obtained 48 h p.i. In B, IFN-β protein was measured at 48 h and was expressed as a ratio of the viral load measured as TCID_50_ units. The data were analyzed using Wilcoxon’s rank sum test.

**Figure 6:**
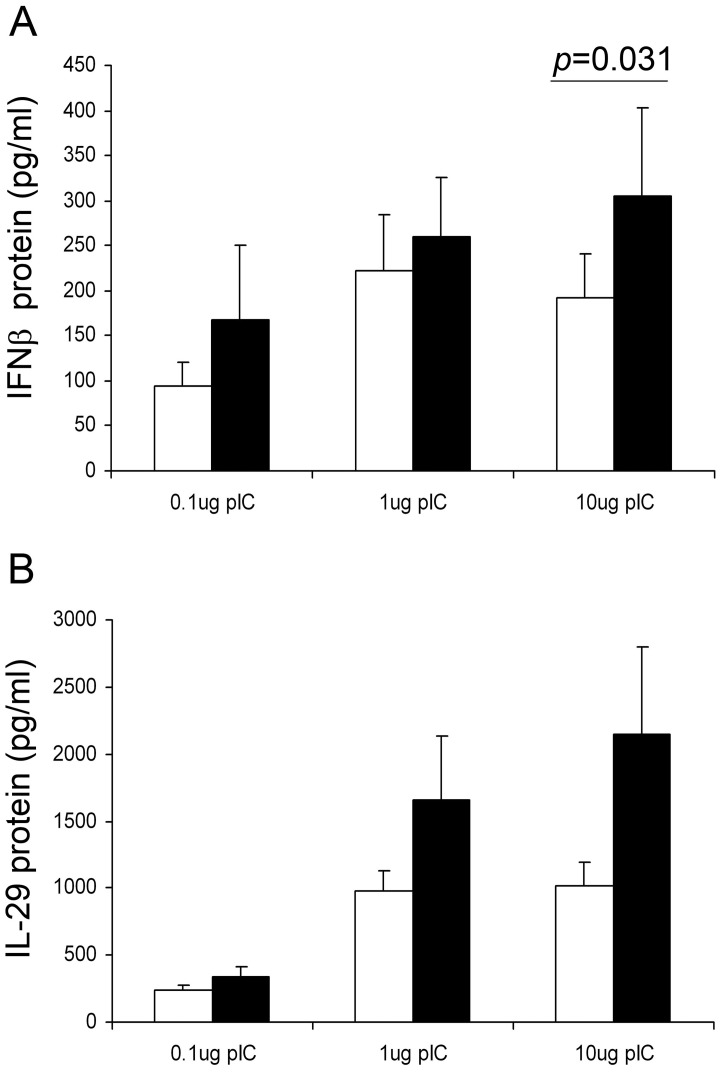
The effect of anti-TGF-β antibodies on release of IFN-β and IFNλ1/IL-29 protein in response to poly IC. PBECs from 6 asthmatic donors were treated with 0.1–10 µg/ml poly IC in the presence of neutralizing anti-TGF-β antibodies (black bars) or an IgG isotype control (open bars) and incubated for 24 h. Supernatants were removed and IFN-β (A) or IFN-λ1/IL-29 protein levels (B) were measured by ELISA. Graphs show (mean±SEM) IFN produced in pg/ml the presence of the control or anti TGF-β antibodies.

### Reverse Transcription and Quantitative Real-time PCR (RT-qPCR)

Total RNAs were extracted from BECs using TRIzol® reagent (Invitrogen, Paisley, U.K.), treated to remove contaminating DNA (DNA-free kit, Ambion, Austin, USA) and cDNA made using Reverse Transcription Kits (PrimerDesign Ltd, Southampton, UK). Expression of RV1B vRNA and mRNAs for human IFN-β, IFN-λ/IL-29, TGF-β_1,_ TGF-β_2_ and TGF-β_3_ was determined using real time quantitative PCR and changes in gene expression expressed relative to the medium control after normalizing to the geometric mean of the housekeeping genes, ubiquitin C (UBC) and glyceraldehyde 3-phosphate dehydrogenase (GAPDH) using the ΔΔCt method. All PCR reagents were purchased from PrimerDesign Ltd, Southampton, UK.

**Figure 7:**
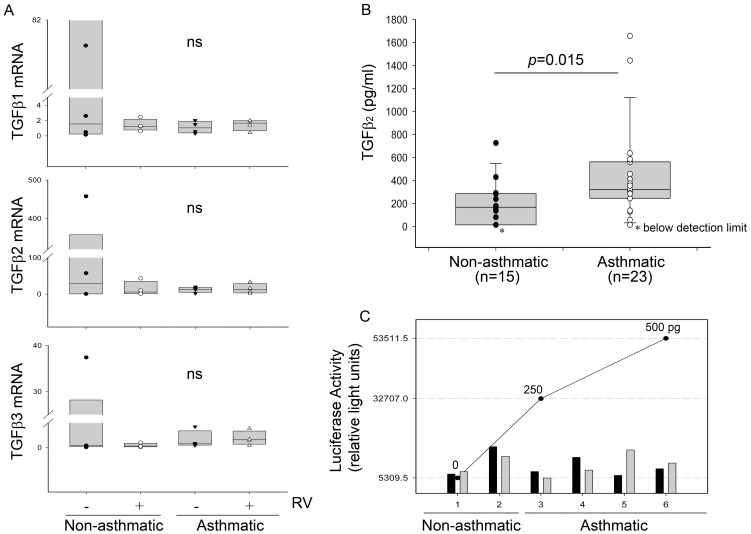
The effect of RV infection on TGF-β isoform expression. TGF-β1, β2, β3 mRNA levels were measured in PBECs from 4 asthmatic and 4 non-asthmatic donors following infection with RV; TGF-β mRNA expression was measured relative to GAPDH/UBC using the ΔΔCt method (A). Total TGF-β_2_ protein levels were measured in conditioned media from PBEC cultures of 15 non-asthmatic and 23 asthmatic donors which were harvested at 48 h. Latent TGF-β_2_ was activated by acid-treatment and total TGF-β_2_ measured by ELISA. Statistical significance was tested using Mann Whitney U test (B). PBEC culture supernatants of 2 non-asthmatic and 4 asthmatic donors were tested in a transformed mink lung cell luciferase bioassay in order to measure active TGF-β in the presence (black bars) or absence (grey bars) of rhinovirus at 5000 TCID_50_ units/10^5^ cells. Superimposed is a standard curve obtained using 250 or 500 pg of active TGFβ (C). Data in panels A and B are given as box and whisker plots showing median, interquartile range and 95% confidence intervals; individual data points are superimposed.

### Interferon Beta (IFN-β), Interferon λ1 (Interleukin-29,IL-29) and TGF-β ELISAs

IFN-β and IL-29/IFN-λ1 release was analyzed using human IFN-β and IFN-λ1/IL-29 ELISA kits (VeriKineTM, NJ, USA) according to manufacturer’s instructions. Latent and active TGF- β_2_ was measured by ELISA (Cat. no. G7600, Promega, Southampton, UK). Cross reactivity with 10 ng/ml TGF-β_1_ was 0.27% and TGF-β_3_ 0.67%. TGFβ_1_ was measured using an Emax Immuno Assay System (Cat# G7590, Promega, Southampton, UK) which shows 1.6% cross-reactivity to 10 ng/ml TGF-β_2_.

**Figure 8:**
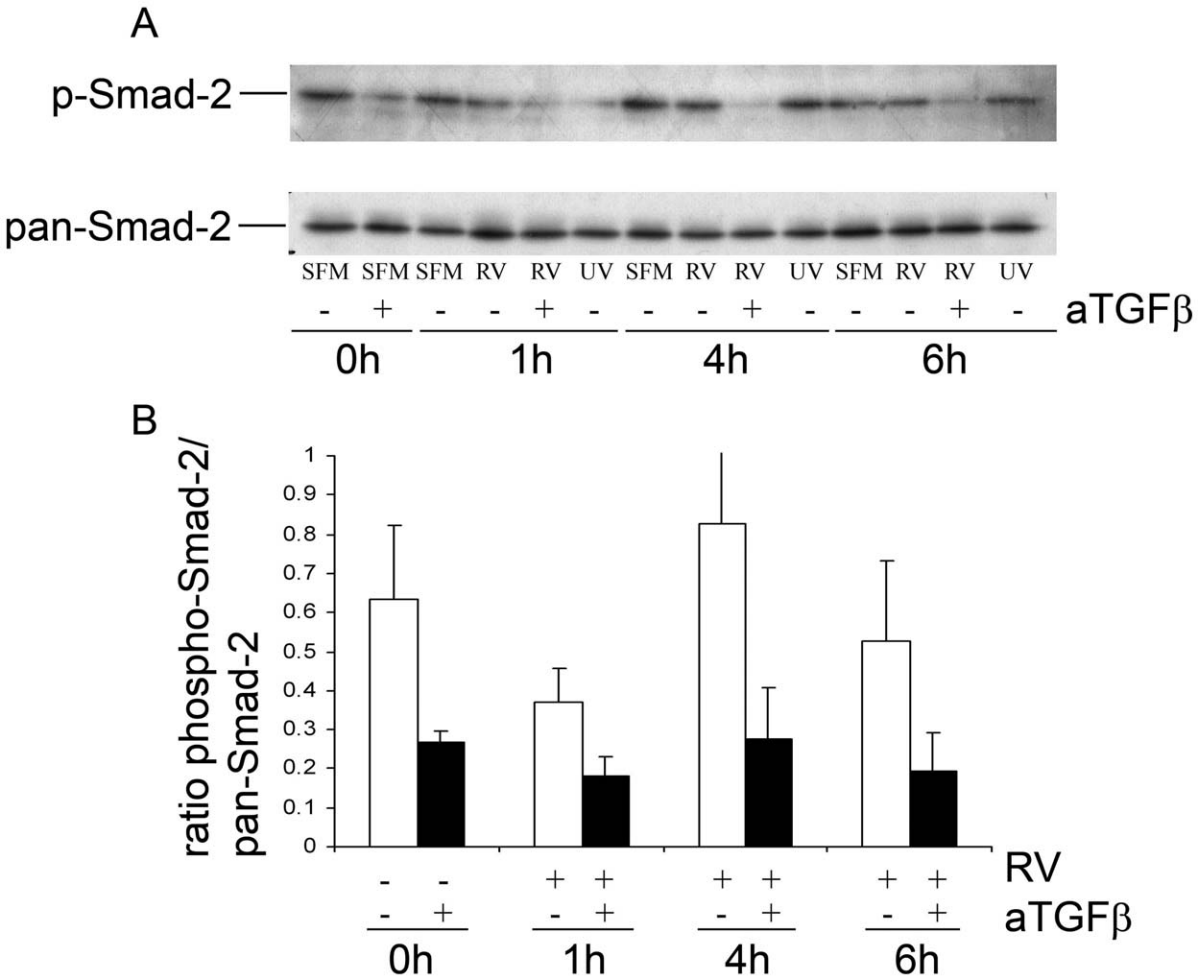
The effect of TGF-β neutralization on basal SMAD2 activation. PBECs from asthmatic donors were treated with RV and anti TGF-β antibody, as indicated, as described in Figure 5. Cell lysates were harvested at 1, 4, and 6 hours post-virus infection and Smad-2 phosphorylation was analysed by Western blotting. A representative Western blot is shown in (A) and densitometric quantification of the experiment repeated using PBECs from 3 different asthmatic subjects is shown in (B).

### Transformed Mink Lung Cell Bioassay

Active TGF-β was measured using a transformed mink lung cell/luciferase bioassay as previously described [16].

**Figure 9:**
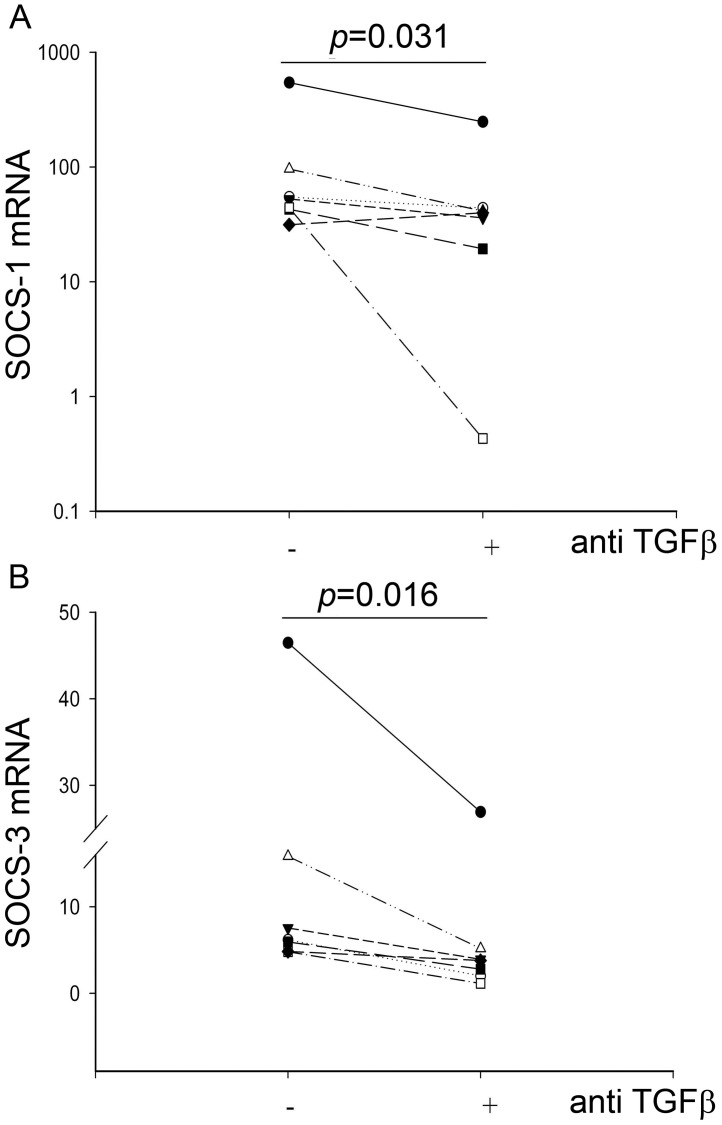
Neutralizing endogenous TGF-β suppresses RV1B mediated SOCS-1 and SOCS-3 gene expression in asthmatic PBECs. Samples were treated as described in Figure 5. SOCS-1 (A) and SOCS-3 (B) gene expression were measured in 7 asthmatic subjects at 48 h p.i. by RT-qPCR and normalized to housekeeping genes. Results were plotted as relative fold-induction using the ΔΔCt method. The Wilcoxon rank sum test was used to analyse statistical significance.

### SDS-PAGE and Western Blotting

PBECs were harvested with lysis buffer and analysed by SDS-PAGE as previously described [17]. Phospho-SMAD-2 and pan-SMAD-2 were purchased from Cell Signalling Biotechnology and used as recommended by the manufacturer.

### Statistical Analysis

Where data were normally distributed, they were analyzed using Student’s paired t test. For non-parametric data, within group comparisons were performed using Wilcoxon Signed Rank test and between group comparisons using Mann-Whitney U test. P<0.05 was considered significant: where statistical significance was observed, p-values are displayed.

## Results

### Exogenous TGF- β_2_ Promotes RV1B Replication and Reduces Type I and Type III Interferon Expression in Response to Viral Infection or Double Stranded RNA

Initial dose response experiments examining the effects of TGF-β_2_ were performed using PBECs from 3 non-asthmatic volunteers. Control experiments established that cell viability was high (>95%) and there were no differences in the levels of cell death (measured as lactate dehydrogenase release) between TGF-β_2_ treated and control cells. Following 24 h pre-incubation in serum-free medium containing 0, 1, 10 or 25 ng/ml TGF-β_2_, cells were then infected with RV1B at 5000 TCID_50_ units/10^5^ cells. Analysis of viral replication at 24 and 48 h post infection revealed a trend for increased viral RNA at 24 h (Figure 1A) and, by 48 h, there was an increase in release of infectious viral particles in the presence of either 10 or 25 ng/ml TGF-β_2_ (7 and 4-fold increase, respectively; Figure 1B). This stimulatory effect was confirmed by testing the effect of TGF-β_2_ (10 ng/ml) pre-treatment on viral replication at 48 h using PBECs from a larger number (n = 10) of non-asthmatic subjects, where a significant increase in viral replication was observed (Figure 1C). A similar increase in viral replication was obtained when TGF-β_2_ was tested using a lower dose (1000 TCID_50_ units/10^5^ cells) of RV1B (p = 0.012, n = 8) (data not shown).

We next analyzed the effect of TGF-β on the innate immune response to RV infection. Measurement of IFN-β protein in culture supernatants of PBECs from non-asthmatic volunteers infected with RV1B for 48 h revealed a significant reduction (p = 0.027) in IFN-β protein levels from infected cells that were pre-treated with TGF-β_2_ compared to those pre-treated in basal medium alone (Figure 2A). IFN-β protein levels were reduced in the presence of TGF-β_2_ in the majority subjects tested. The three subjects showing the largest suppression of IFN-β showed a 1.8–8.1 fold increase in viral replication. In the sole subject that showed an increase in IFN-β expression following TGF-β treatment, we noted that there was a 19-fold increase in viral replication compared with a median 4.4 fold increase. As IFN expression is induced by viral replication, we postulate that in this case, the unusually high increase in viral load eventually over-rode the suppressive effect of TGF-β_2_ to stimulate IFN-β production.

To confirm that the effect of TGF-β_2_ on IFN-β protein levels was not simply a consequence of reduced cell number caused by increased virus-induced cell death, we also measured IFN-β protein in culture supernatants of PBECs treated for 8 h with poly IC, a synthetic TLR-3 ligand that mimics the doubled stranded replicative form of viral RNA and stimulates IFN-β expression without causing significant cell death. We observed approximately 60% reduction in IFN-β protein levels when cultures were treated with poly IC (1 µg/ml) together with either 1 or 10 ng/ml exogenous TGF-β_2_ compared to cultures treated with poly IC alone (p = 0.027 and p = 0.026, respectively) (Figure 2B). Using a higher dose of poly IC (10 µg/ml), we also observed a significant reduction in IFN-β protein when cells were pre-treated with 10 ng/ml TGF-β_2_ (p = 0.028) (Figure 2B). In addition to its effects on Type I IFN production, TGF-β_2_ also caused a significant reduction in Type III IFN expression. Thus, IFN-λ1/IL-29 protein levels were reduced when PBECs were pre-treated with TGF-β_2_ and infected with RV (Figure 3A) or treated with poly IC (Figure 3B). Of note, the one subject that showed an increase in IFN-λ1/IL-29 release following RV and TGF-β_2_ treatment was the same subject that showed an increase in IFN-β production, consistent with the unusually high increase in viral replication observed in this subject.

### Neutralizing TGF-β Bioactivity in Asthmatic and Non-asthmatic PBEC Cultures Resulted in a Greater Reduction of RV1B Replication in Asthmatic PBEC Compared to Non-asthmatic PBECs

To determine whether endogenous production of TGF-β might contribute to the reduced innate immune response of PBECs from asthmatic donors in response to RV infection [4]; [5], we compared the effects of neutralizing antibodies in normal and asthmatic PBECs infected with RV1B. We observed a significantly greater decrease in virus replication at 48 h p.i. when PBECs from asthmatic donors were treated with neutralizing TGF-β antibodies compared to those from non-asthmatic donors (p = 0.02) (Figure 4). Further assessment of the effect of TGF-β neutralization using PBECs from asthmatic donors revealed that at 48 h p.i., the anti-TGF-β antibody caused a significant reduction in release of infectious virus particles (Figure 5A). Since production of IFN-β is dependent on the presence of a ‘danger’ signal (ie. replicating virus), we analyzed IFN-β protein production as a function of viral load. This revealed that in the presence of the TGF-β neutralizing antibody, there was a 3–4 fold increase in IFN-β protein production when expressed as a function of viral load (Figure 5B). To confirm the stimulatory effect of anti TGF-β antibodies on IFN-β production, we also measured IFN-β protein levels following treatment of PBECs from asthmatic donors with poly IC. We found a significant increase in IFN-β protein levels released from cells that were incubated with anti-TGF-β antibody and 10 µg/ml poly IC, compared to when cells were incubated with IgG isotype control and poly IC, p = 0.031 (Figure 6A). We also observed a trend for increased IFNλ1/IL-29 protein levels in the presence of anti-TGF-β antibodies, but this did not reach statistical significance (Figure 6B).

To determine which isoform of TGF-β was contributing to the effect of RV, we analyzed mRNA expression of TGF-β_1_, TGF-β_2_ and TGF-β_3_ following RV infection in asthmatic and non-asthmatic PBECs. Levels of TGF-β_3_ were substantially lower than for TGF-β_1_ and β_2,_ but there was no significant induction of any TGF-β isoform 24 h after RV infection (Figure 7A); further kinetic analysis failed to show an earlier or later effect of infection on TGF-β isoforms expression (data not shown). Even though TGF-β_1_ mRNA was detectable, we did not detect TGF-β_1_ protein by ELISA in conditioned media from either asthmatic or non-asthmatic PBECs. In contrast, TGF-β_2_ protein was not only detectable but was significantly higher in cultures from asthmatic donors compared to those in non-asthmatic donors (p = 0.015) (Figure 7B). However, no active TGF-β_2_ was detected by ELISA. Using a transformed mink lung cell bioassay, we were also unable to detect significant TGF-β activation in the presence of RV (Figure 7C), and levels of activity were low (average <25 pg/ml).

Since infection with RV did not affect expression of any TGF-β isoform, our data suggested that basal endogenous production of TGF-β contributed to the effect on RV infection. Consistent with this, SMAD2 phosphorylation was detectable at baseline in non-infected PBEC cultures and did not increase following RV infection. However, the endogenous phosphorylation of SMAD2 could be blocked by the TGF-β neutralizing antibody (Figure 8A and B).

### Treatment with Anti TGF-β Antibodies Reduces RV-mediated Induction of SOCS-1 and SOCS-3

In order to determine the mechanism by which the presence of endogenous TGF-β suppresses RV1B induced IFN expression, we measured expression of Suppressors of Cytokine Signalling -1 (SOCS-1) and SOCS-3 that interfere with interferon signaling and whose expression has been shown to be induced by TGF-β [18]. In our study, we saw an induction of SOCS-1 and SOCS-3 mRNA in the presence of virus alone in PBECs from asthmatic subjects. When PBECs from asthmatic subjects were treated with anti-TGF-β antibodies prior to virus infection, both SOCS-1 and SOCS-3 mRNA were significantly reduced at 48 h p.i., p = 0.031 and p = 0.016 respectively (Figure 9A and 9B).

## Discussion

In this study, we have demonstrated that a neutralizing antibody that blocks endogenous TGF-β activity significantly reduced RV1B replication in PBECs by increasing IFN expression and reducing SOCS1 and SOCS3 expression. This protection was significantly greater in PBEC cultures from asthmatic donors suggesting that the previously reported susceptibility of asthmatic PBECs to RV infection [4]; [5] may be, in part, due to increased endogenous TGF-β production. We performed our experiments using monolayer cultures, as these cells have a basal cell phenotype and can be used to model areas of damaged/repairing epithelium that are characteristically found in asthmatic airways [10]. Previous studies have shown that basal cells are much more susceptible to viral infection than fully differentiated epithelial cell cultures [19], suggesting that these exposed basal cells will be selectively targeted by RVs that enter the asthmatic lung. However, endogenous TGF-β expression also appears to be important factor that influences the susceptibility of differentiated epithelial cells to RV infection, as we found that neutralization of endogenous TGF-β also suppressed RV replication in air-liquid interface cultures (Figure S1). Our finding that PBECs from asthmatic donors produce more endogenous TGF-β_2_ than PBECs from non-asthmatic donors is consistent with findings of higher levels of TGF-β isoforms in asthmatic mucosa by immunocytochemistry [8]; [15] and the finding that TGF-β_2_ is selectively elevated following allergen challenge [20]. Together, these findings suggest that the presence of elevated levels of TGF-β_2_ in the bronchial epithelium of asthmatic subjects may contribute to virus-induced asthma exacerbations. Furthermore, in addition to endogenously produced epithelial-derived TGF-β_2_, other sources of TGF-β isoforms such as eosinophils whose numbers are increased in asthmatic bronchial epithelium during RV colds and persist during convalescence [21] may also contribute to suppression of the innate immune response to RV infection in asthma.

Analysis of TGF-β isoforms expression revealed that while TGF-β_1_ and TGF-β_2_ mRNAs were readily detectable in PBECs, TGF-β_3_ was close to the limit of detection of our RT-qPCR assay. However, when we examined protein expression, only TGF-β_2_ was detectable and this was present at significantly higher levels in culture supernatants of PBECs from asthmatic donors. Our failure to detect protein expression of TGF-β_1_ even though its mRNA was detectable is consistent with the findings of others [22]. Although we did not investigate the reason for the higher levels of TGF-β_2_ expression by PBECs from asthmatic donors, it has been reported that there are polymorphisms in the *TGFB2* gene promoter that are associated with childhood asthma [23]. In this study, one of the asthma-associated promoter variants, −109 →ACAA ins, was a common variant (allele frequency 0.292) and was shown to increase *TGFB2* promoter reporter activity in the BEAS2B bronchial epithelial cell line. It would therefore be of interest to investigate the *TGFB2* genotype of the donors used in the present study.

TGF-β isoforms are secreted from cells as latent complexes, consisting of mature dimeric growth factor, the latency-associated propeptide (LAP), and latent TGF-β binding protein (LTBP) [24]. Latent TGF-β complexes are normally activated by a diverse group of mechanisms including proteases, thrombospodin-1 (TSP-1), integrins such as α_v_β_6_ and α_v_β_6_, reactive oxygen species (ROS), and low pH [25]. While latent TGF-β_2_ was detectable by ELISA in culture supernatants, we could not detect active TGF-β_2_ in these samples using the same method; we also had limited success in detection of active TGF-β in a sensitive bioassay using transformed mink lung cells. However, there was robust TGF-β activity detected in epithelial cells by phospho-Smad2 immunoblots. Based on our observation that exogenous TGF-β_2_ promoted viral replication, whereas pan-TGF-β neutralizing antibodies markedly suppressed viral replication, and the predominance of TGF-β_2_ at both message and protein level, our data would suggest that active TGF-β_2_ is the predominant isoform promoting viral replication even though it could not be detected by ELISA. The failure to release free active TGF-β into cell supernatants is well described for TGF-β_1_ that requires direct cell-cell contact for its activation [26]. Active TGF-β is also known to have a substantially shorter half-life than the latent form in plasma [27] and several binding proteins such as α2-macroglobulin allow scavenging of active TGF-β from the extracellular space to keep the TGF-β signal local [28]. Furthermore, studies using gene knock out mice have highlighted the roles of the LTBPs in targeting the secreted complex to specific locations in the extracellular matrix [24]; [25]. This ability to target the latent complex in a specific manner may explain why our studies with exogenous TGF-β_2_ required high concentrations of the active TGF-β_2_ to elicit an effect, since appropriate targeting of the growth factor was missing. However, in view of the presence of TGF-β_1_ mRNA expression in the PBECs, we cannot exclude the possibility that low levels of cell-associated active TGF-β_1_ may have been produced that were not detectable as free growth factor in the medium. However, the demonstration of a functional effect of TGF-β_2_ in the absence of free active TGF-β_2_ in cell media raises the intriguing possibility that TGF-β_2_ may be activated by an RGD independent change in its conformational structure.

The pleiotropic effects of TGF-β *in vivo* and *in vitro* provides it with various roles in growth and development, inflammation and repair and host immunity [29]; [30]. One effect of TGF-β is to cause cell cycle arrest and induction of apoptosis *in vitro* through complex signalling pathways involving SMAD proteins [29]. In epithelial cells, TGF-β has been shown to cause G1 cell cycle arrest by activation of anti-proliferative responses such as the transcriptional up-regulation of the cyclin-dependent kinase inhibitors p21Cip1/WAF1 and p15 Ink4b [31]; [32]. Since apoptosis is a key mechanism that limits viral replication [33], it might have been expected that endogenous TGF-β would dampen RV1B replication. However, we could find no effect of anti TGF-β antibodies on caspase activation (Figure S2) and RV1B replication was consistently decreased in the presence of anti TGF-β antibodies. These findings contrast with studies of RSV infection which have reported that exogenous TGF-β_1_ is beneficial for RSV replication [12] via mechanisms that involved cell cycle arrest [34]. Of interest, RSV infection also augmented TGF-β production by infected epithelial cells, although in our own studies, we could find no evidence of increased TGF-β isoform mRNA expression following RV infection. Instead, our data suggested that rather than containing virus infection by inducing apoptosis, the presence of high endogenous level of this cytokine promoted virus replication by suppressing the innate immune response.

In addition to its role in regulating the cell cycle, TGF-β also plays a role in the control of innate and adaptive immunity. Thus, TGF-β plays a role in promotion of Th17 lineage commitment and has been implicated in the initial amplification of the innate immune response through recruitment of monocytes and neutrophils [35], [36]. It also has important anti-inflammatory roles including coordination of regulatory T cell (Treg) development and function including suppression of Th1 and Th2 cell development [30]. While the responses to TGF-β are closely regulated by environmental stimuli and the accompanying cytokine milieu, the extremely pleiotropic nature of this cytokine has led to the suggestion that TGF- β can “act as a light switch: i.e. if it’s on, it will turn it off; if it’s off, it will turn it on [34].” In our experiments, we observed that endogenous TGF-β acts more like an anti-inflammatory cytokine as treatment with neutralizing antibodies promoted induction of both type I and type III interferon responses to either virus infection or the synthetic dsRNA, poly IC. This observation is the first report that TGF-β can directly affect expression of Type III interferon and extends previous studies in bronchial fibroblasts where the authors found a dampening of IFN-β expression following rhinovirus infection in the presence of TGF-β_1_ [14]. However, in the latter case, the authors reported that the effect of TGF-β_1_ appeared to be rapid and mediated via effects on IFN regulatory factor (IRF)-3 pathways [14]. In contrast, in our studies with PBECs, the effect of TGF-β was slow and appeared to involve members of the SOCS family of suppressors of cytokine signaling as evidenced by decreased SOCS-1 and SOCS-3 gene expression when we blocked endogenous TGF-β. SOCS-1 and SOCS-3 do not interfere with direct TLR signaling, but avoid overshooting activation by regulating IFN-β signaling [37] and both have been shown to be induced by TGF-β [18]. Using siRNA targeting SOCS-3, we were able to significantly knockdown SOCS-3 expression and found a trend for increased IFN-β release when epithelial cells were treated with poly IC in the presence of TGF-β (Figure S3). However, as we were unable to significantly knock down SOCS-1 using the same approach, we were unable to test the cumulative effect of attenuating the inhibitory effects of both suppressor proteins. Thus, while further work is still required to demonstrate causality between SOCS-1 and -3 and modulation of the IFN response, the slow kinetics of the anti TGF-β effect on viral replication are more consistent with a slow, cumulative effect of TGF-β involving increased SOCS expression and suppression of IFN-β signalling rather than blockade of the initiating signal involving IRF-3. Of further note is the occurrence of genetic polymorphism in the *SOCS-1* gene where a particularly significant association has been found between asthma susceptibility and a promotor polymorphism (-1478CA) which was shown to cause increased transcription of SOCS-1 [38]. Thus, there is a potential for gene-gene interactions involving polymorphisms in the *TGFΒ2* and *SOCS1* genes linked to virus-induced asthma exacerbations.

In conclusion, our data suggest that higher levels of endogenous TGF-β expression contribute to the decreased innate immune response to virus infection in asthmatic epithelial cells. This involved reduction of *both* IFN-β and IFNλ1/IL-29 mRNA and protein expression in response to RV infection and was mirrored by similar responses when the cells were exposed to the TLR3 agonist, poly IC. This response was associated with higher endogenous levels of TGF-β_2_ protein and elevated SOCS1 and SOCS3 expression that could be down-regulated by TGF-β neutralizing antibodies. Although not investigated directly, our data suggest that genetic polymorphisms in either the *TGFΒ2* or *SOCS-1* gene may dictate varying degrees of susceptibility to virus infection in asthma. There is also the possibility that production of other isoforms of TGF-β by cell types such as eosinophils, whose numbers are increased in asthmatic bronchial epithelium during rhinovirus colds and persist during convalescence [21], may also directly contribute to virus-induced exacerbations by affecting the ability of epithelial cells to mount an adequate innate anti-viral immune response.

## Supporting Information

Figure S1 The effect of neutralizing anti-TGF-β antibodies on rhinovirus replication in PBECs grown on air-liquid interface (ALI). PBECs from 5 subjects were differentiated at an air-liquid interface for 3 weeks, as previously described (Xiao et al. J Allergy Clin Immunol 128, 549–556, 2011). Cells were then pre-treated apically and basolaterally for 24 h with anti-TGF-β or an isotype control (10 µg/ml), followed by RV1B infection (MOI = 5) for 6 h. The virus was then washed off and cells were further incubated for 18 or 42 h in the presence of anti-TGF-β (aTGFb) or an isotype (IgG1) control. Apical washes were analysed for the release of viral particles as TCID_50_/ml after 24 h (A) (n = 5) or 48 h (B) (n = 4) from the start of infection.
(DOCX)

Figure S2 Caspase 3/7 activity of RV1B-infected PBEC in the presence or absence of TGF-β. PBECs from a healthy donor were seeded into a collagen-coated 96-well plate and incubated overnight at 37°C. Cells were then pre-treated with TGF-β_2_ (10 ng/ml) and incubated for 24 hrs after which they were infected with RV1B (MOI = 0.05) for 1 hour, washed, and further incubated in media for 4, 8, and 24 hrs in the absence or presence of TGF-β_2_. After each time point, a luminogenic caspase-3/7 substrate was added to each sample and incubated for 1 hour. Luminescence was measured on a TopCount plate reader.
(DOCX)

Figure S3 The effect of SOCS-3 knockdown on IFN-β protein in TGF-β treated PBECs. PBECs were transfected with 100 nM siRNA targeted against SOCS-3 (SOCS-3) or a negative control siRNA (Neg) for 24 h followed by treatment with 1 µg/ml poly IC for 8 hours in the presence or absence of 10 ng/ml TGF-β_2_. A: Cell conditioned media were analysed for secreted IFN-β protein; the data are expressed as a percent of cells treated with the Negative control siRNA and poly IC in the absence of TGF-β (n = 4). B: SOCS-3 mRNA expression was determined by RT-qPCR. There was significant suppression of SOCS-3 expression in the presence of SOCS-3 siRNA compared with control (P<0.02)
(DOC)
