# Supplementary material for: Transforming Growth Factor-Beta Promotes Rhinovirus Replication in Bronchial Epithelial Cells by Suppressing the Innate Immune Response
Source: PLoS One. 2012 Sep 6;7(9):e44580. doi: 10.1371/journal.pone.0044580 (PMC3435262; doi:10.1371/journal.pone.0044580)
Supplement: Figure S1 — The effect of neutralizing anti-TGF-β antibodies on rhinovirus replication in PBECs grown on air-liquid interface (ALI). PBECs from 5 subjects were differentiated at an air-liquid interface for 3 weeks, as previously described (Xiao et al. J Allergy Clin Immunol 128, 549–556, 2011). Cells were then pre-treated apically and basolaterally for 24 h with anti-TGF-β or an isotype control (10 µg/ml), followed by RV1B infection (MOI = 5) for 6 h. The virus was then washed off and cells were further incubated for 18 or 42 h in the presence of anti-TGF-β (aTGFb) or an isotype (IgG1) control. Apical washes were analysed for the release of viral particles as TCID50/ml after 24 h (A) (n = 5) or 48 h (B) (n = 4) from the start of infection. (DOCX) [file pone.0044580.s001.docx]

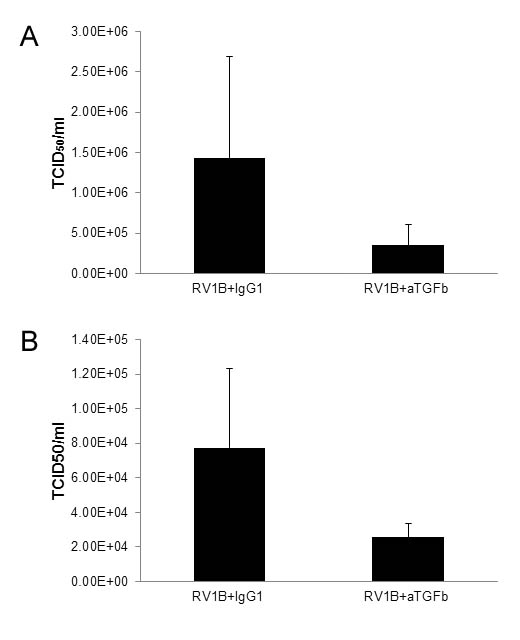


*Online Supplement Figure S1*

The effect of neutralizing anti-TGF-β antibodies on rhinovirus replication in PBECs grown on air-liquid interface (ALI). PBECs from 5 subjects were differentiated at an air-liquid interface for 3 weeks, as previously described (Xiao et al. J Allergy Clin Immunol 128, 549–556, 2011). Cells were then pre-treated apically and basolaterally for 24h with anti-TGF-β or an isotype control (10μg/ml), followed by RV1B infection (MOI=5) for 6h. The virus was then washed off and cells were further incubated for 18 or 42h in the presence of anti-TGF-β (aTGFb) or an isotype (IgG1) control. Apical washes were analysed for the release of viral particles as TCID_50_/ml after 24h (A) (n=5) or 48h (B) (n=4) from the start of infection.
