# Supplementary material for: Transforming Growth Factor-Beta Promotes Rhinovirus Replication in Bronchial Epithelial Cells by Suppressing the Innate Immune Response
Source: PLoS One. 2012 Sep 6;7(9):e44580. doi: 10.1371/journal.pone.0044580 (PMC3435262; doi:10.1371/journal.pone.0044580)
Supplement: Figure S2 — Caspase 3/7 activity of RV1B-infected PBEC in the presence or absence of TGF-β. PBECs from a healthy donor were seeded into a collagen-coated 96-well plate and incubated overnight at 37°C. Cells were then pre-treated with TGF-β2 (10 ng/ml) and incubated for 24 hrs after which they were infected with RV1B (MOI = 0.05) for 1 hour, washed, and further incubated in media for 4, 8, and 24 hrs in the absence or presence of TGF-β2. After each time point, a luminogenic caspase-3/7 substrate was added to each sample and incubated for 1 hour. Luminescence was measured on a TopCount plate reader. (DOCX) [file pone.0044580.s002.docx]

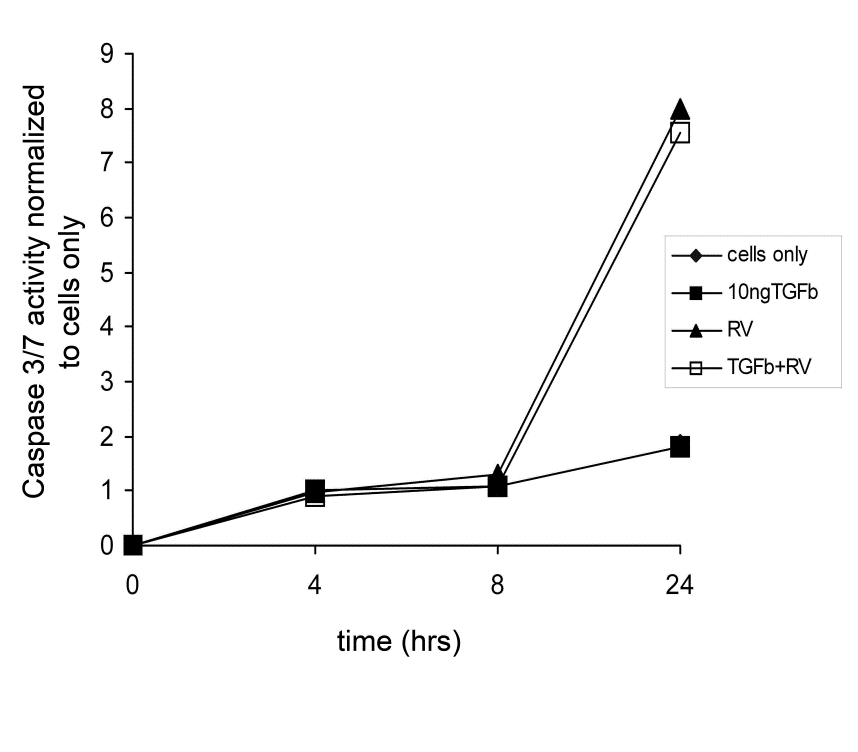


*Online Supplement Figure S2*

Caspase 3/7 activity of RV1B-infected PBEC in the presence or absence of TGF-β. PBECs from a healthy donor were seeded into a collagen-coated 96-well plate and incubated overnight at 37^º^C. Cells were then pre-treated with TGF-β_2_ (10 ng/ml) and incubated for 24 hrs after which they were infected with RV1B (MOI=0.05) for 1 hour, washed, and further incubated in media for 4, 8, and 24 hrs in the absence or presence of TGF-β_2_. After each time point, a luminogenic caspase-3/7 substrate was added to each sample and incubated for 1 hour. Luminescence was measured on a TopCount plate reader.
