# Supplementary material for: Transforming Growth Factor-Beta Promotes Rhinovirus Replication in Bronchial Epithelial Cells by Suppressing the Innate Immune Response
Source: PLoS One. 2012 Sep 6;7(9):e44580. doi: 10.1371/journal.pone.0044580 (PMC3435262; doi:10.1371/journal.pone.0044580)
Supplement: Figure S3 — The effect of SOCS-3 knockdown on IFN-β protein in TGF-β treated PBECs. PBECs were transfected with 100 nM siRNA targeted against SOCS-3 (SOCS-3) or a negative control siRNA (Neg) for 24 h followed by treatment with 1 µg/ml poly IC for 8 hours in the presence or absence of 10 ng/ml TGF-β2. A: Cell conditioned media were analysed for secreted IFN-β protein; the data are expressed as a percent of cells treated with the Negative control siRNA and poly IC in the absence of TGF-β (n = 4). B: SOCS-3 mRNA expression was determined by RT-qPCR. There was significant suppression of SOCS-3 expression in the presence of SOCS-3 siRNA compared with control (P<0.02) (DOC) [file pone.0044580.s003.doc]

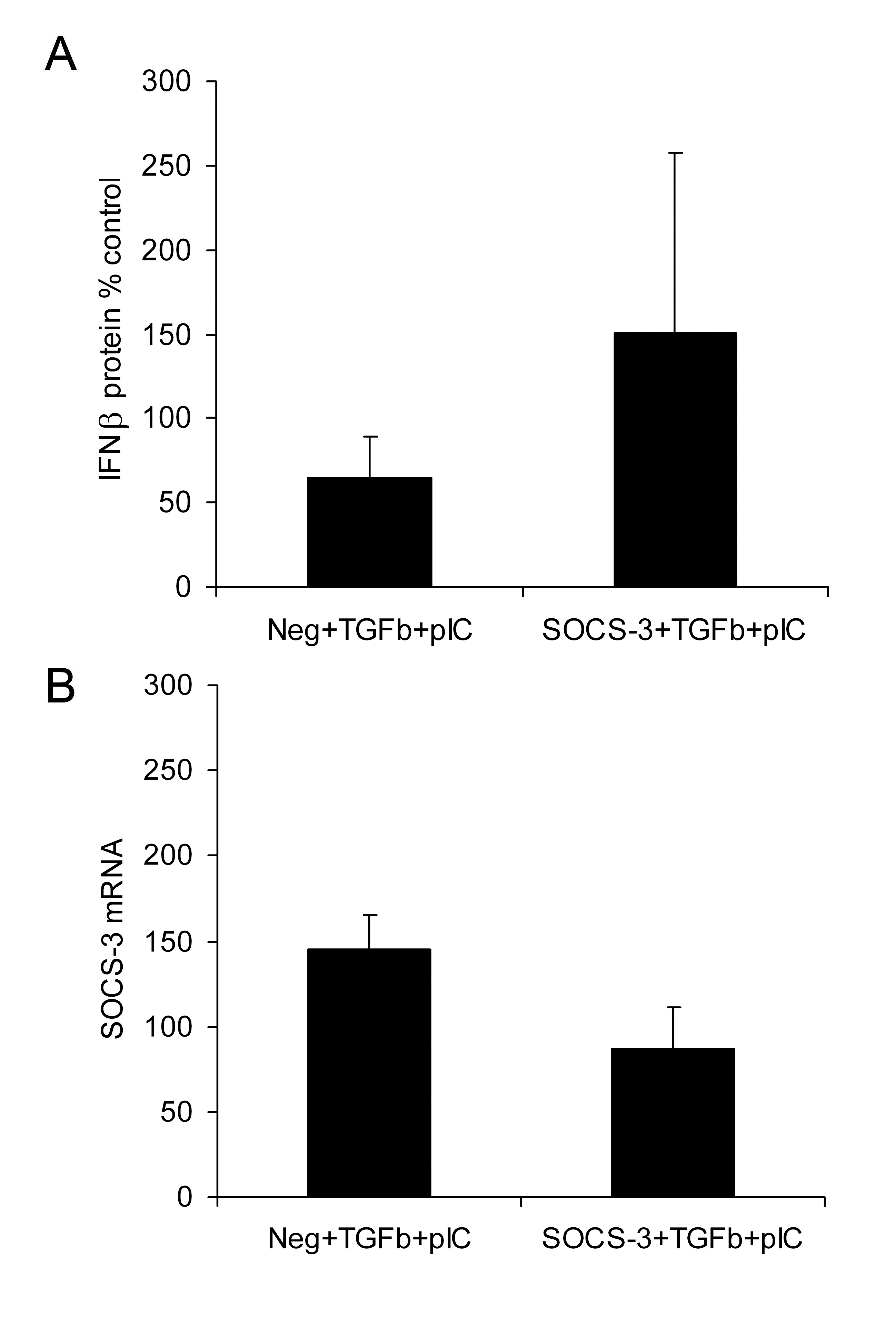


*Online Supplement Figure S3*

The effect of SOCS-3 knockdown on IFN-β protein in TGF-β treated PBECs. PBECs were transfected with 100 nM siRNA targeted against SOCS-3 (SOCS-3) or a negative control siRNA (Neg) for 24h followed by treatment with 1 µg/ml poly IC for 8 hours in the presence or absence of 10 ng/ml TGF-β_2_.

A: Cell conditioned media were analysed for secreted IFN-β protein; the data are expressed as a percent of cells treated with the Negative control siRNA and poly IC in the absence of TGF-β (n=4).

B: SOCS-3 mRNA expression was determined by RT-qPCR. There was significant suppression of SOCS-3 expression in the presence of SOCS-3 siRNA compared with control (P<0.02)
